# Supplementary material for: Derivation and Validation of a Predictive Score for Respiratory Failure Worsening Leading to Secondary Intubation in COVID-19: The CERES Score
Source: J Clin Med. 2022 Apr 13;11(8):2172. doi: 10.3390/jcm11082172 (PMC9028352; doi:10.3390/jcm11082172)
Supplement: Supplementary file 1 [file jcm-11-02172-s001.zip › jcm-1677220-supplementary/Figure S1.pdf]

Figure S1

	**OR (95% CI)**
**Age**	**1.05 (1.01 - 1.11)**
BMI	0.96 (0.88 - 1.03)
SAPS2	0.97 (0.92 - 1.02)
SOFA	1.04 (0.98 - 1.1)
CT-scan extension	0.99 (0.97 - 1.02)
**FiO2**	**1.03 (1.01 - 1.07)**
VWF:Ag	1 (1 - 1)
Angiopoietin 2	1 (1 - 1)
VEGF	1 (0.99 - 1)
Syndecan-1	1 (1 - 1)
**Endocan**	**1.1 (1.02 - 1.24)**
suPAR	1.17 (1 - 1.41)
PAI-1	1 (0.99 - 1.01)
TFPI	1 (1 - 1.01)
CRP	1 (1 - 1.01)
PCT	1.17 (1 - 1.79)
LDH	1 (1 - 1)
ALAT	1 (0.98 - 1.01)
ASAT	1.01 (1 - 1.02)
Bilirubin	1.13 (0.92 - 1.38)
Creatinine	1.01 (0.98 - 1.04)
Ferritin	1 (1 - 1)
TQ ratio	0.67 (0.14 - 1.51)
Fibrinogen	0.81 (0.58 - 1.1)
D-dimers	1.06 (1.01 - 1.16)
Hemoglobin	0.98 (0.78 - 1.24)
Leucocytes	0.99 (0.9 - 1.09)
Neutrophiles	1.02 (0.91 - 1.13)
Lymphocytes	1.18 (0.74 - 2.06)
**Platelets**	**0.99 (0.98 - 1)**

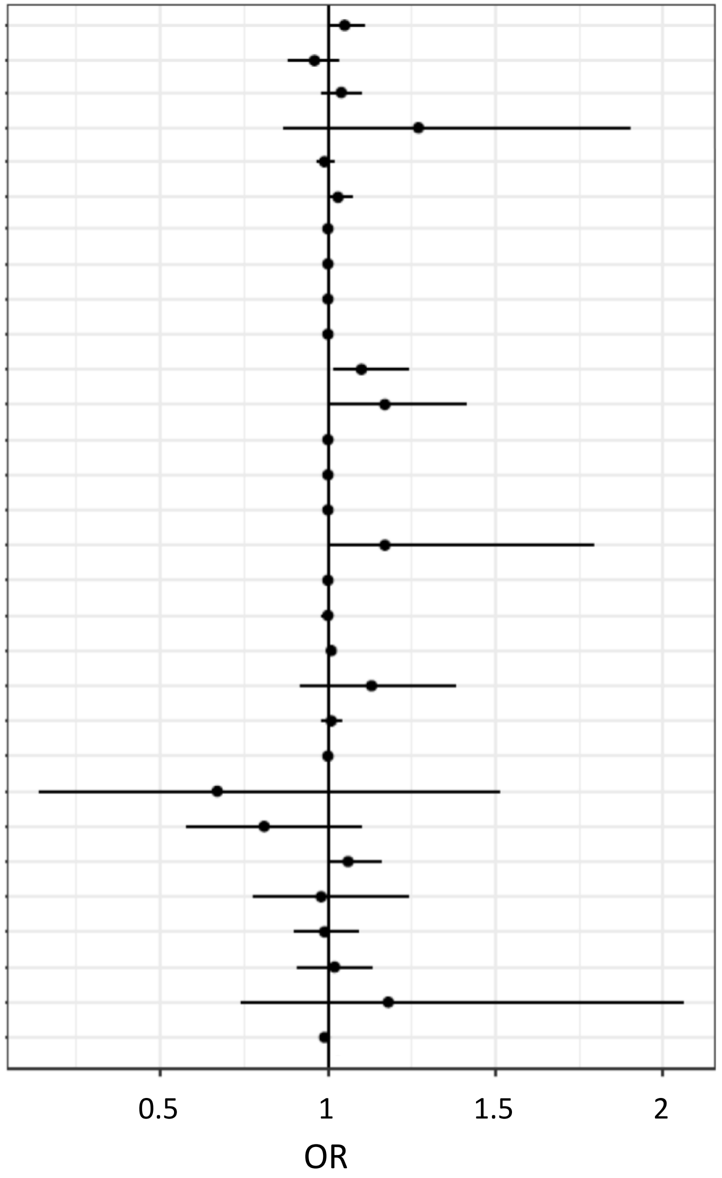

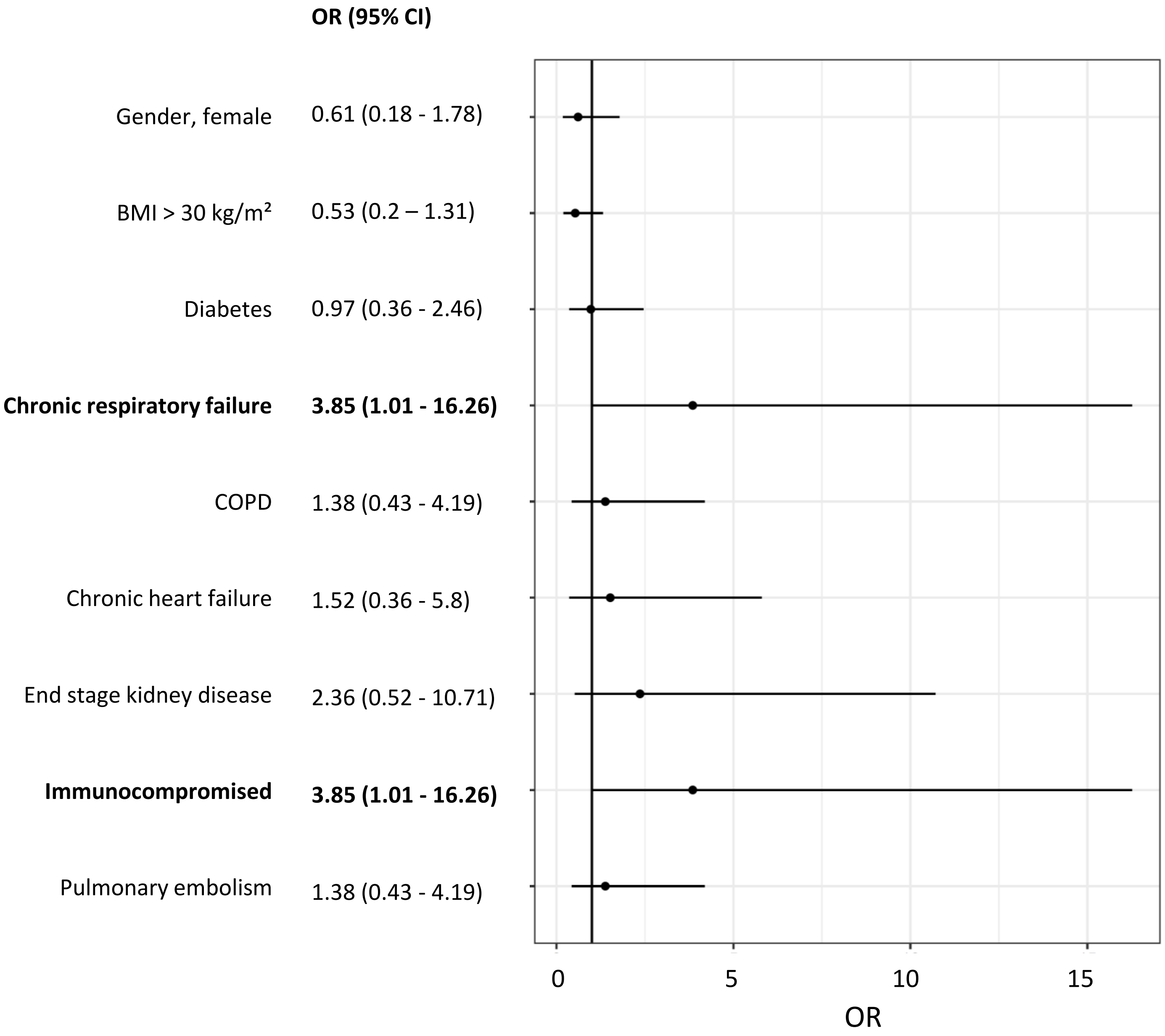

OR (95% CI)
Gender, female
0.61 (0.18 - 1.78)
BMI > 30 kg/m²
0.53 (0.2 – 1.31)
Diabetes
0.97 (0.36 - 2.46)
Chronic respiratory failure
3.85 (1.01 - 16.26)
COPD
1.38 (0.43 - 4.19)
Chronic heart failure
1.52 (0.36 - 5.8)
End stage kidney disease
2.36 (0.52 - 10.71)
Immunocompromised
3.85 (1.01 - 16.26)
Pulmonary embolism
1.38 (0.43 - 4.19)
0
5
10
15
OR

Figure S1. Univariate logistic regressions in the derivation cohort for continuous and categorical variables on ICU admission. Results are presented as OR with 95% CI for late worsening of acute respiratory failure within 15 days following ICU admission. Numbers and variables are indicated in bold characters in case of p-values < 0.05. BMI—body mass index, COPD—chronic obstructive pulmonary disease, CT—computed tomography, FiO2—fraction of inspired oxygen, ICU—intensive care unit, OR—odds ratio, PAI-1—plasminogen activator inhibitor-1, SAPS 2—simplified acute physiology score 2, SOFA—sequential organ failure assessment, suPAR—soluble urokinase plasminogen activator receptor, TFPI—tissue factor pathway inhibitor, VEGF—vascular endothelial growth factor, VWF:Ag—Von Willebrand factor antigen.
