# Supplementary material for: Derivation and Validation of a Predictive Score for Respiratory Failure Worsening Leading to Secondary Intubation in COVID-19: The CERES Score
Source: J Clin Med. 2022 Apr 13;11(8):2172. doi: 10.3390/jcm11082172 (PMC9028352; doi:10.3390/jcm11082172)
Supplement: Supplementary file 1 [file jcm-11-02172-s001.zip › jcm-1677220-supplementary/Figure S2.pdf]

Figure S2

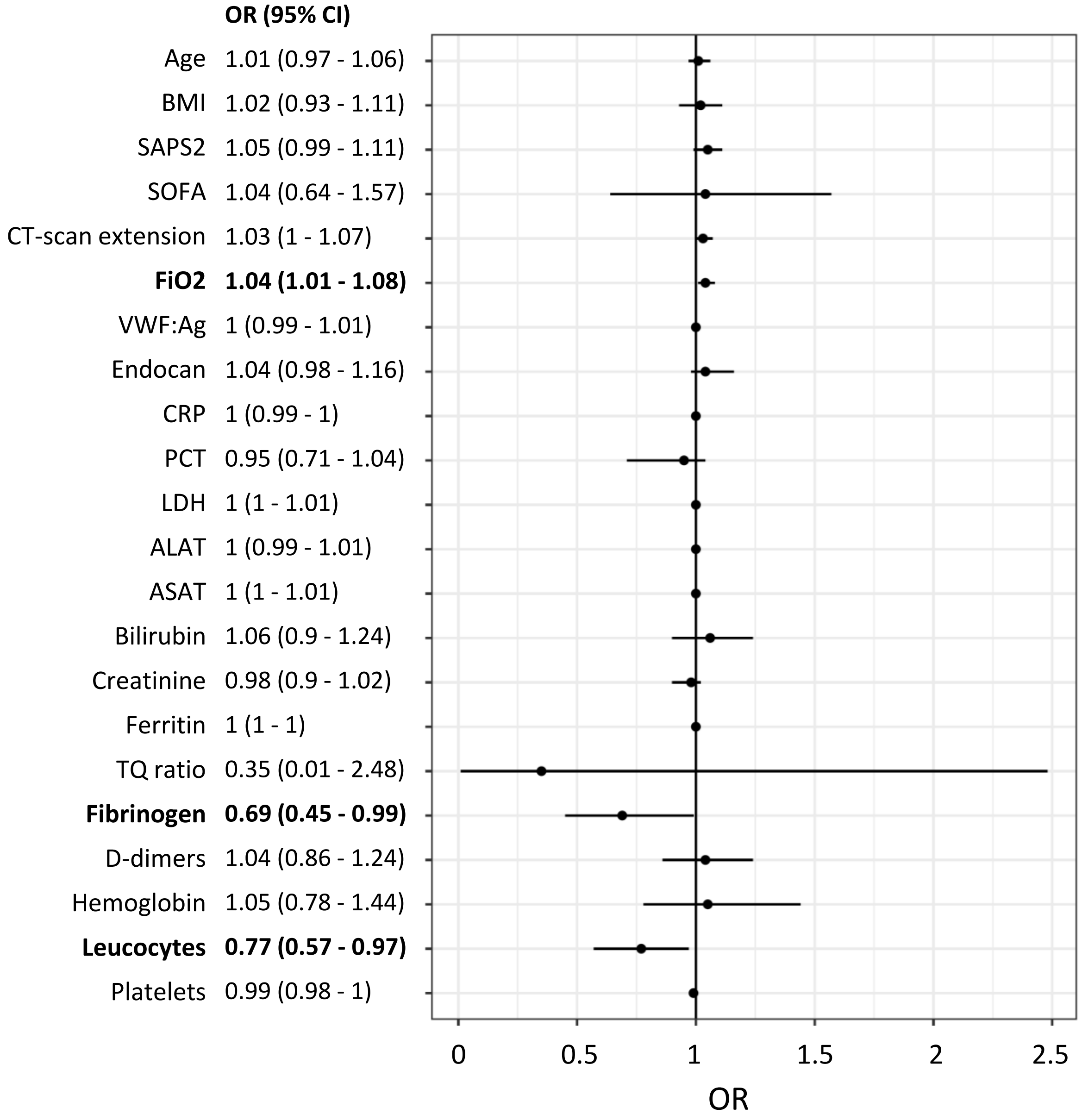
OR (95% CI)
Age 1.01 (0.97 - 1.06)
BMI 1.02 (0.93 - 1.11)
SAPS2 1.05 (0.99 - 1.11)
SOFA 1.04 (0.64 - 1.57)
CT-scan extension 1.03 (1 - 1.07)
FiO2 1.04 (1.01 - 1.08)
VWF:Ag 1 (0.99 - 1.01)
Endocan 1.04 (0.98 - 1.16)
CRP 1 (0.99 - 1)
PCT 0.95 (0.71 - 1.04)
LDH 1 (1 - 1.01)
ALAT 1 (0.99 - 1.01)
ASAT 1 (1 - 1.01)
Bilirubin 1.06 (0.9 - 1.24)
Creatinine 0.98 (0.9 - 1.02)
Ferritin 1 (1 - 1)
TQ ratio 0.35 (0.01 - 2.48)
Fibrinogen 0.69 (0.45 - 0.99)
D-dimers 1.04 (0.86 - 1.24)
Hemoglobin 1.05 (0.78 - 1.44)
Leucocytes 0.77 (0.57 - 0.97)
Platelets 0.99 (0.98 - 1)
0
0.5
1
1.5
2
2.5
OR

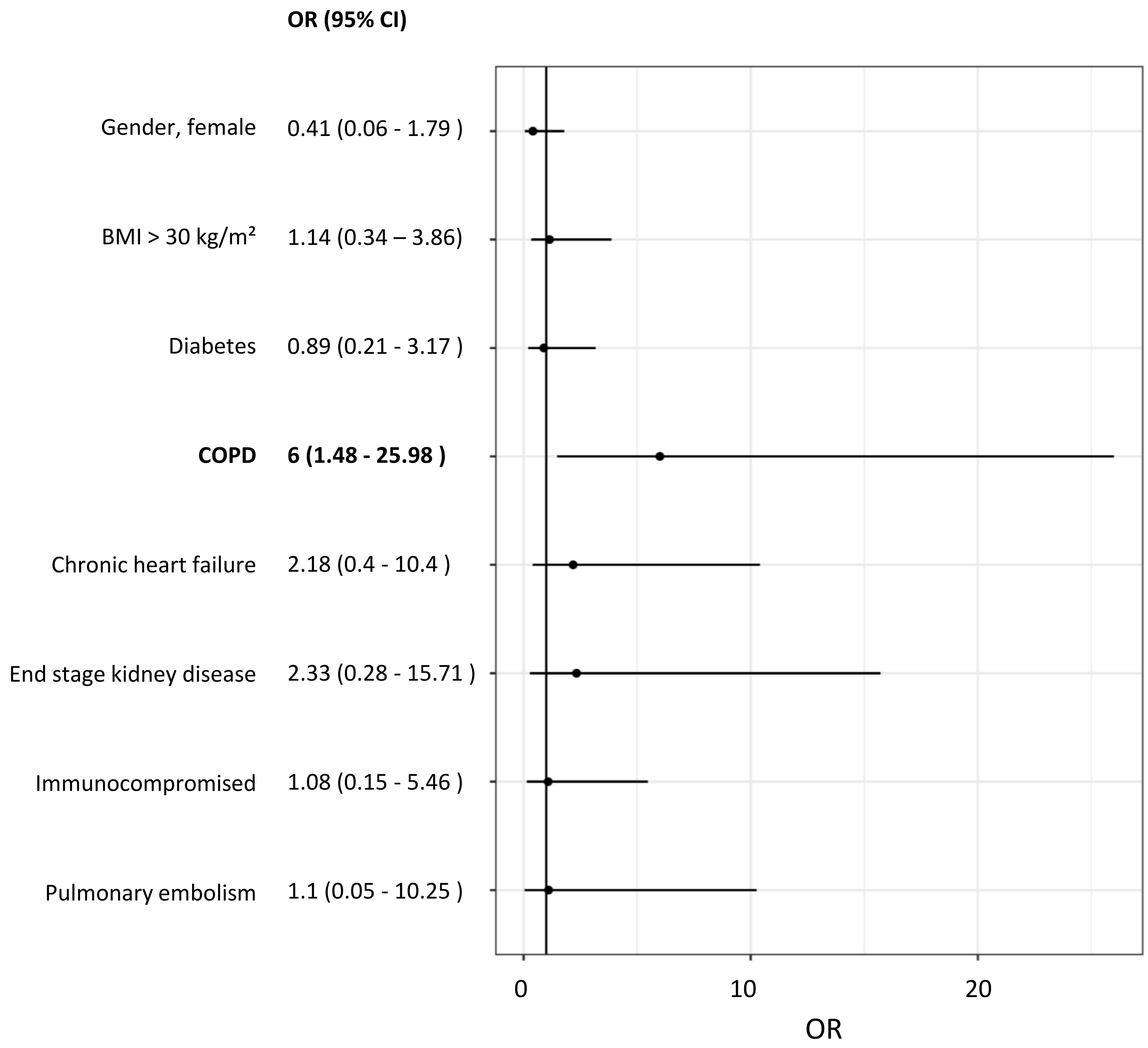
OR (95% CI)
Gender, female
0.41 (0.06 - 1.79)
BMI > 30 kg/m²
1.14 (0.34 – 3.86)
Diabetes
0.89 (0.21 - 3.17)
COPD
6 (1.48 - 25.98)
Chronic heart failure
2.18 (0.4 - 10.4)
End stage kidney disease
2.33 (0.28 - 15.71)
Immunocompromised
1.08 (0.15 - 5.46)
Pulmonary embolism
1.1 (0.05 - 10.25)
0
10
20
OR

Figure S2. Univariate logistic regressions in the validation cohort for continuous and categorical variables on ICU admission. Results are presented as OR with 95% CI for late worsening of acute respiratory failure within 15 days following ICU admission. Numbers and variables are indicated in bold characters in case of p-values < 0.05. BMI—body mass index, COPD—chronic obstructive pulmonary disease, CT—computed tomography, FiO2—fraction of inspired oxygen, ICU—intensive care unit, PAI-1—plasminogen activator inhibitor-1, OR—odds ratio, SAPS 2—simplified acute physiology score 2, SOFA—sequential organ failure assessment, suPAR—soluble urokinase plasminogen activator receptor, TFPI—tissue factor pathway inhibitor, VEGF—vascular endothelial growth factor, VWF:Ag—Von Willebrand factor antigen.
