# Supplementary material for: Derivation and Validation of a Predictive Score for Respiratory Failure Worsening Leading to Secondary Intubation in COVID-19: The CERES Score
Source: J Clin Med. 2022 Apr 13;11(8):2172. doi: 10.3390/jcm11082172 (PMC9028352; doi:10.3390/jcm11082172)
Supplement: Supplementary file 1 [file jcm-11-02172-s001.zip › jcm-1677220-supplementary/Table S1.pdf]

Table S1. Counts of missing data for continuous variables

Variables	Missing data	
	Derivation cohort (N = 92)	Validation cohort (N = 59)
Age	0 (0)	0 (0)
BMI	0 (0)	1 (2)
SAPS2	0 (0)	0 (0)
SOFA	0 (0)	0 (0)
CT-scan extension	27 (29)	14 (24)
FiO2	0 (0)	0 (0)
VWF:Ag	0 (0)	0 (0)
Angiopoietin 2	0 (0)	--
VEGF	0 (0)	--
Syndecan-1	0 (0)	--
Endocan	0 (0)	0 (0)
suPAR	0 (0)	--
PAI-1	1 (1)	--
TFPI	0 (0)	--
CRP	0 (0)	0 (0)
PCT	4 (4)	3 (5)
LDH	14 (15)	16 (27)
ALAT	1 (1)	0 (0)
ASAT	1 (1)	0 (0)
Bilirubin	1 (1)	0 (0)
Creatinine	0 (0)	1 (2)
Ferritin	13 (14)	9 (15)
TQ ratio	0 (0)	1 (2)
Fibrinogen	0 (0)	0 (0)
D-dimers	0 (0)	3 (5)
Hemoglobin	0 (0)	0 (0)
Leucocytes	0 (0)	1 (2)
Neutrophiles	16 (17)	24 (41)
Lymphocytes	16 (17)	24 (41)
Platelets	0 (0)	0 (0)

Table S1. Counts of missing data for continuous variables. Results are presented as number (%). BMI Body mass index, CT Computed tomography, FiO2 fraction of inspired oxygen, PAI-1 Plasminogen activator inhibitor-1, SAPS2 Simplified acute physiology score 2, SOFA Sequential organ failure assessment, suPAR Soluble urokinase plasminogen activator receptor, TFPI Tissue factor pathway inhibitor, VEGF Vascular endothelial growth factor, VWF:Ag Von Willebrand factor antigen.
